# American Surgery in Paris—Letter from Dr. John Swinburne

**Published:** 1871-04

**Authors:** 


### American Surgery in Paris—Letter from Dr. John Swinburne.

FROM THE MEDICAL AND SURGICAL REPORTER.

[We present the following extracts from a letter of Dr. JOHN SWINBURNE, dated Paris, Feb. 27th, 1871, addressed to Dr. Wm. BAILEY, of Albany. Dr. Swinburne has been in Paris during the siege, attached to the American Ambulance Corps.—EDS.]

* * * We have had the good luck to treat three cases of compound fracture of the thigh, and all recovered with good limbs. We have here almost the only surviving amputation of the thigh in Paris. I understand the success outside of Paris has been bad. The result of these three cases of compound fracture of the thigh more than compensate me for all the deprivations, trouble, and time spent here during the siege.

Of the first sixty-two soldiers received into the ambulance only two died, and the immediate cause of their death was tetanus. Four of the above number were amputated through the thigh for wounds in the knee joint. Two compound fractures of the thigh, one of the neck, and one of the middle of the shaft, have been success fully treated by conservation, and are now well, walking with crutches, and with straight limbs. One compound fracture of the tibea, just below the joint, recovered finely, but owing to some unfortunate accident, by which he seriously injured the thigh, he subsequently died. Also, two compound fractures of the wrist and two of the ankle joint recovered with useful limbs. Two compound and comminuted fractures of the scapula recovered. Also a number of compound fractures of the forearm, fibula, feet, hands, besides one resection of the shoulder joint, in a soldier who was suffering from a large pleuritic effusion. * * * *

JOHN SWINBURNE, M. D.

